# Are positive changes in potential determinants associated with increased fruit and vegetable intakes among primary schoolchildren? Results of two intervention studies in the Netherlands: The Schoolgruiten Project and the Pro Children Study

**DOI:** 10.1186/1479-5868-5-21

**Published:** 2008-04-25

**Authors:** Nannah I Tak, Saskia J te Velde, Johannes Brug

**Affiliations:** 1 EMGO-institute, VU University Medical Centre, Amsterdam, The Netherlands; 2 Department of Public Health, Erasmus University Medical Center, Rotterdam, The Netherlands

## Abstract

**Background:**

To investigate if positive changes or maintenance high scores on potential behavioral determinants of fruit and vegetable (F&V) intake are associated with increased or maintenance favorable levels of F&V intake frequency in the same time lapse or later in time. Data were used from two intervention studies in the Netherlands: the Schoolgruiten Project and the Pro Children Study.

**Methods:**

A design with baseline and two follow-up measurements. 344 children of the Dutch Schoolgruiten Project and 258 children of the Pro Children Study completed questionnaires, including questions on general demographics, usual F&V intake frequency, important potential determinants of F&V intake, such as taste preferences of F&V, availability of F&V, knowledge of recommended intake levels of F&V, self-efficacy for eating F&V, and parental influences for eating F&V. Three different associations between changes in determinants of F&V intake and changes in F&V intake frequency were assessed by multilevel multinomial regression analyses.

**Results:**

Results of one of the investigated associations indicated that in both studies behavior change (increase in F&V intake frequency) was preceded by changes in the following variables; liking of fruit, parental facilitation of vegetables, family rules for eating vegetables and availability at home of vegetables. Furthermore, changes in F&V intake frequency preceded changes in liking of F&V later in time.

**Conclusion:**

In accordance with behavior change theories, the present study provides some evidence that behavior change was preceded by changes in certain potential determinants of F&V intake. Potential determinants of F&V intake that appear to be important to induce behavior change were liking of fruit, parental facilitation of vegetables, family rules for eating vegetables and availability at home of vegetables. Some evidence was also found that behavior changes may precede changes in presumed determinants of F&V intake, such as liking of F&V.

## Background

Ample intake of fruit and vegetables (F&V) is part of dietary recommendations in many countries. However, among schoolchildren across Europe, the reported intake of F&V is lower than recommended [1]. The Dutch recommendations for F&V intake for 10–12-year-old children are to eat at least two pieces of fruit (about 200–250 grams) and 150–200 grams vegetables per day [2].

According to health behavior change theories such as the Social Cognitive Theory and [3] the Theory of Planned Behavior [4], increasing F&V intake can be induced by changes in presumed behavioral determinants, such as attitude, social influence and self-efficacy or behavioral control [5,6]. Furthermore, studies on determinants of F&V intake among children showed that taste preference, availability, parental intake levels, and knowledge of recommended intake levels are of additional potential importance as mediators or determinants of behavior and behavior change [7-12]. However, the majority of these studies applied cross-sectional designs, which does not allow concluding upon causal relationships between potential determinants and F&V intake. It might as well be that changes in F&V intake precede changes in presumed determinants. For instance, increased exposure to F&V can influence taste preferences [13-15].

Other studies conducted mediation analyses to study whether an intervention effect could be explained by presumed mediating variables (which are often the most important potential determinants of the behavior in question) [16]. These mediation analyses aim at explaining the effect of an intervention and often study changes in behavior and changes in mediating variables occurring in the same time period. Therefore, strictly speaking, these studies cannot draw conclusions regarding the direction of the relationship between the potential determinants or mediators and behavior or behavior change. Longitudinal studies are therefore required to better understand the relationships between potential important determinants of F&V intake and F&V intake among children. Results should than be used to further improve ongoing and newly developed intervention programs that often claim to aim at behavior change by changing important potential determinants of F&V intake [17-21].

**Figure 1:**
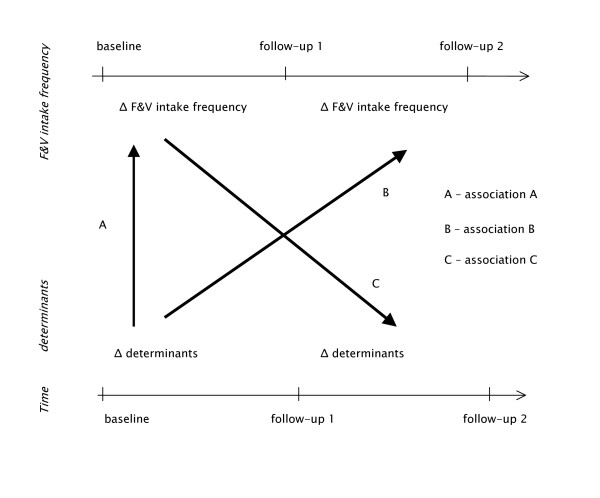
Design of the study with the three assessed associations between changes in important determinants and changes in F&V intakes.

Data of the Dutch Pro Children Study and the Schoolgruiten Project provide the opportunity to study changes in F&V intake frequency and potential determinants measured at three different time points (see Figure 1). Therefore, the aim of the present study was to investigate whether positive changes in or maintaining of high scores on the presumed important determinants of F&V intake in the first time lapse (period between baseline and first follow-up) were associated with positive changes or maintenance of favorable levels in F&V intake frequency in the same time lapse (association A in Figure 1) and with positive changes or maintenance of favorable levels in F&V intake frequency later in time (association B in Figure 1). Furthermore, we examined whether positive changes or maintenance of favorable levels in F&V intake frequency were associated with positive changes in or maintenance of high scores on the variables that were identified as potentially important determinants of F&V intakes in earlier studies, later in time (association C in Figure 1). These analyses were conducted separately for the Dutch Schoolgruiten Project and the Dutch Pro Children Study. We hypothesized that those who reported positive changes or kept a favorable score in a presumed determinant of F&V, also reported positive changes or kept favorable levels of F&V intake frequency (in the same time period or later in time).

## Methods

### Description of both projects

The Pro Children Study is a cross- European study on F&V intake among primary schoolchildren (age; 10–11 years old at baseline) [22]. For the present analyses, we only included the Dutch intervention part of the Pro Children Study, which was implemented in Rotterdam, one of the major cities in the Netherlands.

The Pro Children Study addressed a wide range of important determinants of F&V intake based on a previously published theoretical framework [22]. This theoretical framework recognizes the role of the physical environment, such as the availability and accessibility of foods [19], as well as social environmental factors [9]. Among children, the role of family environment is of specific importance. Furthermore, children spend a considerable amount of their time at school, and the school environment may also importantly influence nutrition and physical activity behaviors [13].

The Schoolgruiten Project is also a Dutch intervention study among primary schoolchildren (age; 9–10 years old at baseline). This project was implemented in two intervention cities in the Netherlands: in The Hague, one of the major cities in the west part of the Netherlands, and in Almelo, a medium sized city in the east part. Similar to the Pro Children Study, the main strategy within the Schoolgruiten Project was targeting taste preferences, availability and accessibility.

For the present study, we only included children from the intervention schools, since these children are more likely to show changes in potential determinants of F&V intake, as a consequence of the intervention activities [20,21]. The data was used as observational longitudinal cohort data.

### Design of the studies

The baseline survey of the Pro Children Study was conducted in September 2003. First follow-up was performed nine months after the baseline measurement (May 2004) and second follow-up was performed exactly one year after first follow-up (May 2005).

The baseline survey of the Schoolgruiten Project was conducted in The Hague in the spring of 2003 and in Almelo in the autumn of 2003. First follow-up was conducted in both cities exactly one year later and second follow-up was conducted exactly two years later.

During the intervention period the intervention schools of the Pro Children Study were provided with a piece of fruit or ready-to-eat vegetables (cherry tomatoes, baby carrots) for free during a fruit break twice a week. In addition, a classroom curriculum was implemented, which consisted of worksheets and a web-based computer tailored feedback tool [23]. Furthermore, parents were encouraged to be involved in the project by means of their children's homework assignments, parental newsletters, and a parent version of the web-based computer tailored tool that enabled them to get personalized feedback on their own F&V intake levels.

The children of the intervention schools of the Schoolgruiten Project received also a piece of fruit or ready-to-eat vegetables (cherry tomatoes, baby carrots) for free during a fruit break twice a week. All children ate the piece of fruit or vegetable in their own classroom. Apart from increasing availability and accessibility, this F&V scheme was also supposed to increase the children's exposure to F&V, which in turn can influence the children's taste preferences [15].

Additionally, a school curriculum, developed and carefully pre-tested by the Netherlands Nutrition Center Foundation, aiming at increasing knowledge and skills related to F&V intake was offered to the intervention schools. The intervention schools were not obliged to use this curriculum, but they were strongly encouraged to do so.

Schoolgruiten Project was approved by the Netherlands Organization for Health Research and Development (ZonMw) Program for Prevention and by The World Cancer Research Fund. The Pro Children Study was approved by the medical ethical committee of the Erasmus University Medical Center. For the Pro Children Study, the parents provided written informed consent for themselves and their child. For the Schoolgruiten Project the informed consent was authorized by a legal representative (the school board).

### Recruitment of the schools and study sample

For the Pro Children Study, 76 primary schools were initially sampled of which 24 schools with 735 eligible students (both intervention and control schools) agreed to participate in the total study. These 24 schools were randomly assigned to an intervention (12 schools) or a control group (12 schools).

As mentioned before, only the data of the twelve intervention schools are included in the present analyses. At the start of the study, 410 pupils were eligible for participation. Because of absence at the day of data collection or because of lack of informed consent (n = 41), 369 children actively participated at baseline. Since one of the study purposes was to investigate whether positive changes in or maintenance of high scores on the potential determinants of F&V intake were associated with positive changes or maintenance of favorable levels in F&V intake frequency later in time, only the children that had valid data on all three measurements were included. A total of 258 children were finally included for analyses.

Children with complete self-reported data on F&V intake frequency at baseline but not at first or at second follow-up were considered as dropouts. Dropout was due to children who moved to other places or schools, did not graduate to the next grade, were absent on the day of administration at first or at second follow-up (n = 87), or had missing F&V reports at one of the three measurements moments (n = 24).

The Schoolgruiten Project included 31 intervention schools. All fourth grades from primary schools in the intervention cities were eligible for participation and schools were randomly approached by phone, and invited to participate in this survey. All children (n = 693) who were present on the day of administration completed the questionnaires. Two schools were not willing to participate anymore at first follow-up, resulting in fewer children (n = 613) at first follow-up. Six schools were not willing to participate anymore at second follow-up, again reducing the number of children (n = 504) in the sample. Only the children that completed all questions on fruit intake frequency or all questions on vegetable intake at all three measurements were included in the analyses. Finally, a total of 344 children were included in the analyses.

Again, children with valid self-reported data on fruit and/or vegetable intake frequency at baseline but not at first or at second follow-up were considered as dropouts. Dropout was due to the loss of eight schools (n = 146), because children moved to other places or schools, did not graduate to the next grade, were absent on the day of administration at first or at second follow-up (n = 190), or had missing F&V reports at one of the three measurements moments (n = 13).

Since some children had missing data for some of the variables, the number differed slightly between different analyses, as indicated in the relevant tables.

### Procedure

In both studies schoolchildren received questionnaires, which they completed during one-school hour. Questionnaire administration was according to a written protocol. In the Schoolgruiten project, questionnaire administration was led by the teacher, while in the Pro Children Study children completed the questionnaires in the presence of a project worker.

Children of both studies received another questionnaire to take home for completion by one of their parents. Responses were treated anonymously and confidential.

All data of the Pro Children Study were entered and cleaned in the national centers according to a standardized protocol. All national data sets were pooled and further data processing and quality control was carried out in the Data Management Center at the University of Vienna (for more information on protocols and data management, as well as the questionnaires visit the website of Pro Children [24]).

### Questionnaires

For both projects two questionnaires were developed, a parental and a child version. In the Pro Children Study the questionnaires were pilot-tested for validity and reliability. Specific information on the development, reliability and validity of the questionnaires has been published previously [25-27]. Briefly, correlations between the frequency questionnaire answers regarding total F&V intake and the reference method (a 7-day food record) varied between 0.38 and 0.53. Correlations for total F&V intake between the test and the re-test measurement varied between 0.47 and 0.76 [26].

The internal consistency of the scales and the test-retest reliability and predictive validity of the behavior theory-based constructs measuring the personal, social and environmental correlates of F&V intake of the Pro Children questionnaire is measured in five European countries in 10–11-year-old children [27]. The test-retest reliability was good to very good (intra-class correlation coefficient (ICC > 0.60) for 12 out of the 15 fruit constructs and also 12 out of the 15 vegetable constructs. Acceptable ICCs, ranging between 0.50 and 0.59, were found for the remaining constructs. Cronbach's α values were moderate to high (range 0.52 to 0.89) with the exception of the general self-efficacy scale, which had a value below 0.50 for both fruit (α = 0.42) and vegetables (α = 0.49). Spearman correlations with intake ranged between -0.16 and 0.54 for personal determinants and between 0.05 and 0.38 for environmental determinants. Compared with other studies, predictive validity can be considered moderate to good [27].

The child and parent questionnaires developed for the Schoolgruiten Project were based on the two Pro Children questionnaires. A more detailed description of the questions and answer alternatives of the Schoolgruiten questionnaire has been published previously [28].

For both studies, child reported F&V intake frequency and their potential determinants were used. For both studies, general demographic information, like information on the parent's country of birth, level of education and child's age, was retrieved from the parent's questionnaire.

### F&V intake frequency

Both the Pro Children Study and the Schoolgruiten Project used food frequency questions to assess usual frequency of intake of F&V. This measure is very useful and often used in studies on correlates, predictors or determinants of food intake. This measure assesses individual usual intake frequency. More specific measures such as 24 H recall methods do not provide valid assessments of usual intake.

In the Pro Children Study, usual daily intake frequency of F&V was assessed with different food frequency questions [26]. Frequency of fruit intake was assessed by one question: "How often do you usually eat fresh fruit". Frequency of salad and grated, other raw and cooked vegetables intake was measured separately by three questions with eight response alternatives ranging from "never" (0) to "every day, more than twice per day" (7). Mean intake in grams per day was calculated by the sum of frequency of intake of salad/grated, raw and cooked vegetables multiplied by a standard portion size (60 gram for cooked vegetables, 40 gram for salad, and 50 gram for raw vegetables [26]). This questionnaire is validated by a 7-day food record; the first day was a weighed record and the following 6 days were estimated records [26].

In the Schoolgruiten Project, we calculated average daily fruit intake (in pieces per day) as the number of days when fruit was eaten multiplied by the number of pieces eaten per day, divided by seven. We calculated average daily vegetable intake (in grams per day) as the number of days when vegetables were eaten multiplied by the amount of vegetables shown on the indicated photographs, divided by seven. The photographs were based on the validated Dutch EPIC Food Frequency Questionnaire [29]. The pictures represent a combination of standard portions, four plates with standard portions of boiled vegetables, and four plates with standard portions of composite dishes. A composite dish contains at average a third of mixed vegetables [30]. For calculations we combined these portion sizes by adding up these amounts and dividing by two. For calculation of raw vegetable consumption, 35 gram was considered a standard child portion size. This is half of the standard adult portion size (70 gram) according to the Netherlands Nutrition Center Foundation [30]. Total frequency of vegetable intake was the sum of boiled and raw vegetables. A more detailed description of the questions and answer alternatives has been published elsewhere [28].

### Potential determinants of F&V intake

In the Pro Children Study a wide range of potential personal, social and environmental determinants related to F&V intake were measured [27]. For the present study we made a selection of these variables that were measures in the two studies, based on the literature and previous results of cross-sectional multivariate associations reported for the Pro Children Study [10,12]. In these studies the authors concluded that liking of F&V, knowledge of recommended intake levels of F&V, self-efficacy for eating F&V, availability of F&V at home, and parental influences were the most important potential determinants of F&V intake. Therefore, we included these variables in the present study.

All these potential determinants were measured separately for both F&V intakes. All factors, except knowledge of recommended intake levels, were assessed using a 5-point Likert scale: fully disagree (-2) to fully agree (+2). To assess knowledge of recommended intake levels, children were asked on an eight-point scale how much fruit or vegetables they should eat every day. Response options ranged from "no fruit or no vegetables" (0) to "5 pieces or portions per day or more" (7). This was subsequently recoded into a dichotomous variable (less than the recommended intake levels versus the recommended intake levels or more).

Also in the Schoolgruiten Project different potential determinants of fruit intake were measured. Based on the same arguments as described before, we included the following determinants in the current study: liking and knowledge of recommended intake levels, both for F&V intakes, and accessibility and availability at home, only for fruit intake. All these determinants were assessed with questions similar to those used in the Pro Children Study [27].

### General demographic information

For both studies, distinctions were made between children of Dutch, non-Western, and non-Dutch Western ethnicity (Europe (excluding Turkey), North America, Oceania, Indonesia or Japan), according to the definition of the Dutch Institute of Statistics [31]. When at least one of the parents was born in a non-Western country the child was considered as of non-Western ethnicity.

Family educational level was used as a measure of socio-economic position. For both studies, parents responded to questions regarding their educational level. Educational level was treated as a categorical variable, using three categories based on the highest educational level of one of the parents (primary school or pre-vocational training = low; high school or medium level vocational training = medium; high level vocational training, college or university training = high).

### Statistical analyses

Means, standard deviations and percentages were calculated to describe the key variables.

Selective dropout was assessed by logistic regression analyses with gender, parental educational level, ethnicity, region of residence of the children (only for Schoolgruiten study) (categorical variables), and intake frequency of fruit or vegetable at baseline (continuous variables) as independent variables and dropout (1 = yes, 0 = no) as the dependent variable.

As suggested by Twisk and Proper, associations between changes in potential determinants and changes in F&V intake frequency were assessed by means of multilevel multinomial logistic regression analyses [32]. This method takes into account that change can either be increase or decrease, or no change (stable). Furthermore, it accounts for the phenomenon that children with high intake levels at baseline, are less likely to increase their intake, and are more likely to report less extreme values at follow-up (i.e. regression to the mean) [32]. For these analyses newly constructed categorical dependent variables were created, describing change in a specific determinant. The categories were: the 'decreasers' group (= the reference group (0)), the 'stable low' (SL) group (1) and the 'stable high' (SH) group, which was merged with the increasers group (2). These two groups were merged together because both outcomes were a positive outcome. To describe the positive change or maintenance of favorable levels in fruit or vegetable intake frequency in the first and second time lapse, we used a relative measure, to overcome the phenomenon that children of this study tended to overestimate their FV intake frequency at baseline, as published previously [28]. The phenomenon of over-reporting by younger children was also observed by Reinaerts *et al*. [33]. For the relative measure, we constructed quartiles of intake frequency at all time points and analyzed whether children changed their relative position. This resulted in a dichotomous variable: 'the SL' and 'the decreasers' (0), (negative outcome) and 'the SH' and 'the increasers' (1) (positive outcome). All children who complied with the Dutch daily recommendations for fruit or vegetables intake were also assigned to the 'SH/increasers' group, because these outcomes were still a positive outcome.

A multilevel analysis was used to take into account the nested design of the study (pupils were nested within schools). Analyses were further adjusted for children's age, gender, parental education level, ethnicity, and region of residence (only for the Schoolgruiten study).

According to the aims of the present study, we performed a series of analyses (see Figure 1) assessing; A) if positive changes or maintenance of favorable levels of F&V intake frequency in the first time lapse, was associated with higher odds of having positively changed or kept high scores of the specific potential determinants in the same time lapse (association A in Figure 1); B) if positive change of maintenance of favorable levels of F&V intake frequency later in time was associated with higher odds of having positively changed or kept high scores of the specific potential determinants in the previous time lapse (association B in Figure 1); 3) if positive changes or keeping high scores of the specific potential determinants later in time were associated with higher odds of having increased F&V intake frequency in the previous time lapse (association C in Figure 1).

Associations were estimated by odds ratios (ORs), which reflect the odds for the group that increased or maintained favorable levels of F&V intake frequency of being in the specific category (for change in determinant) compared to being in the reference category (= decreasers group). When cells for the multinomial logistic regression analyses include a small number, no reliable ORs can be estimated. Five percent of the total sample or less was considered as a small number and in that case three categories were merged into two categories to solve this problem.

The data analyses were performed using SPSS 11.0 (SPSS Inc., Chicago, IL, USA, 1999). The multi-level analyses were conducted using MLwiN software (Version 2.01) [34]. The significance level was set at p < 0.05.

## Results

### Dropout

There was no selective dropout in the Pro Children Study. For the Schoolgruiten Project selective dropout was found for boys (OR = 1.83, 95% CI 1.25 – 2.68), and for those residing in Almelo, the eastern region (OR = 2.47, 95% CI 1.62 – 3.78), due to the loss of eight schools in this region.

### Characteristics

Slightly more girls than boys participated in both studies and the majority of the children were from non-western ethnicity in the Schoolgruiten Project (Table 1). At baseline, the age of all children of both studies ranged between 8.5 – 12.1 years.

**Table 1:** Characteristics of the children of the Schoolgruiten Project and the Pro Children Study at baseline

**Characteristics**		**Schoolgruiten Project**		**Pro Children Study**	
		N	Mean (SD) or %	N	Mean (SD) or %
Age of the children, years		344	10.0 (0.6)	255	10.7 (0.5)

Gender	Boys	147	42.7	104	40.3
	Girls	197	57.3	154	59.7

Ethnicity	Native Dutch children	133	38.7	112	48.9
	Children of Western ethnicity	18	5.2	16	7.0
	Children of non-Western ethnicity	193	56.1	101	44.1

Educational level of the parents	Low	108	35.9	94	43.5
	Moderate	108	35.9	60	27.8
	High	85	28.2	62	28.7

### F&V intake frequency

Table 2 shows the observed mean values for the intake frequency of F&V at baseline, at first and at second follow-up for the children of both studies. The table also shows the number of children that increased or kept their relative high intake levels based on quartiles of intake levels. The cut-off points of the quartiles of the F&V intakes frequency for both studies are provided in Table 3.

**Table 2:** F&V intakes frequency at baseline, at first and at second follow-up, separately for the children of the Schoolgruiten Project and for the children of the Pro Children Study

**F&V INTAKES**	**N**	**Measurements**		
		
		Baseline	First follow-up	Second follow-up
**Schoolgruiten Project**				

Reports on fruit intake frequency (pieces per day) (Mean (SD))	327	1.74 (1.12)	1.64 (0.97)	1.52 (0.91)
Number (%) of increasers/stable high fruit intake frequency ^1^	327	-	172 (52.6)	180 (55.0)
Reports on vegetable intake frequency (gram per day) (Mean (SD))	291	113.3 (60.3)	111.4 (55.3)	102.6 (46.4)
Number (%) of increasers/stable high vegetable intake frequency ^1^	291	**-**	155 (53.3)	158 (54.3)

**Pro Children Study**				

Reports on fruit intake frequency (pieces per day) (Mean (SD))	258	1.16 (0.93)	1.21 (0.92)	1.15 (0.89)
Number (%) of increasers/stable high fruit intake frequency ^1^	258	-	129 (50.0)	147 (57.0)
Reports on vegetable intake frequency (gram per day) (Mean (SD))	258	80.7 (64.7)	85.7 (56.2)	76.7 (52.0)
Number (%) of increasers/stable high vegetable intake frequency ^1^	258	-	146 (56.6)	146 (56.6)

^1 ^Based on the relative measure of F&V intake frequency (quartiles, see Table 3)

**Table 3:** Cut-off points of the quartiles of the F&V intakes frequency, at baseline, at first and at second follow-up, separately for the children of the Schoolgruiten Project and the Pro Children Study

	First quartile	Second quartile	Third quartile	Fourth quartile
**Schoolgruiten Project**				

Reports on fruit intake frequency (pieces per day), at baseline	0 – 0.86	0.87 – 1.43	1.44 – 2.14	2.15 – 4.00
Reports on fruit intake frequency (pieces per day), at first follow-up	0 – 0.86	0.87 – 1.43	1.44 – 2.00	2.01 – 4.00
Reports on fruit intake frequency (pieces per day), at second follow-up	0 – 1.00	1.01 – 1.43	1.44 – 2.00	2.01 – 4.00
Reports on vegetable intake frequency (gram per day), at baseline	0 – 71.0	71.1 – 100.3	100.4 – 149.8	149.9 – 288.0
Reports on vegetable intake frequency (gram per day), at first follow-up	19.1 – 71.0	71.1 – 100.3	100.4 – 141.5	141.6 – 288.0
Reports on vegetable intake frequency (gram per day), at second follow-up	17.9 – 71.0	71.1 – 96.0	96.1 – 121.5	121.6 – 288.0

**Pro Children Study**				

Reports on fruit intake frequency (pieces per day), at baseline	0 – 0.43	0.44 – 1.00	1.01 – 2.00	2.01 – 3.00
Reports on fruit intake frequency (pieces per day), at first follow-up	0 – 0.43	0.44 – 0.79	0.80 – 2.00	2.01 – 3.00
Reports on fruit intake frequency (pieces per day), at second follow-up	0 – 0.43	0.44 – 1.00	1.01 – 2.00	2.01 – 3.00
Reports on vegetable intake frequency (gram per day), at baseline	0 – 39.6	39.7 – 68.9	69.0 – 101.0	101.1 – 400.0
Reports on vegetable intake frequency (gram per day), at first follow-up	0 – 49.6	49.7 – 75.7	75.8 – 112.8	112.9 – 301.8
Reports on vegetable intake frequency (gram per day), at second follow-up	0 – 46.5	46.6 – 64.2	64.3 – 99.9	100.0 – 450.0

### Analyses of relation A (see Figure 1)

The children who increased or kept their relatively high fruit intake frequency in the first time lapse were more likely to have increased their liking of fruit and increased their perceptions of availability at home of fruit in the same time lapse. We found this association for both studies (see Table 4). We found this association in the Pro Children Study also for general self-efficacy for eating fruit, parental active encouragement to eat fruit, and the family rule demanding the child to eat fruit.

**Table 4:** Likelihood of change in determinants in the first time lapse and change in F&V intake frequency in the same time lapse, and later in time estimated with multinomial multilevel analyses, separately for children of the Schoolgruiten Project and the Pro Children Study

**Change in determinants of F&V intake in first time lapse**		**Stable high/increase in fruit intake frequency in first time lapse**			**Stable high/increase in vegetable intake frequency in first time lapse**			**Stable high/increase in fruit intake frequency in second time lapse**			**Stable high/increase in vegetable intake frequency in second time lapse**		
				
**Schoolgruiten Project**													
				
		**N**	**OR**	**95% CI**	**N**	**OR**	**95% CI**	**N**	**OR**	**95% CI**	**N**	**OR**	**95% CI**
Liking	Decreased (2)	-	-	-	43	1.00	-	-	-	-	43	1.00	-
	Stable low (1) (decreased (1))	68	1.00	-	98	1.03	0.61 – 1.73	68	1.00	-	98	0.76	0.46 – 1.26
	Stable high – increased (0)	**220**	**2.89**	**1.64 – 5.09**	**111**	**4.14**	**2.44 – 7.02**	**220**	**1.93**	**1.10 – 3.40**	111	1.30	0.79 – 2.14
Knowledge	Decreased/stable low (1)	91	1.00	-	176	1.00	-	91	1.00	-	176	1.00	-
	Stable high – increased (0)	172	1.64	0.97 – 2.79	76	1.59	0.88 – 2.87	172	0.88	0.52 – 1.50	76	1.43	0.79 – 2.58
Taking fruit with-out asking	Decreased/stable low (1)	57	1.00	-	-	-	-	57	1.00	-	-	-	-
	Stable high – increased (0)	225	1.18	0.63 – 2.18	-	-	-	225	1.21	0.65 – 2.24	-	-	-
Availability at home	Decreased/stable low (1)	55	1.00	-	-	-	-	55	1.00	-	-	-	-
	Stable high – increased (0)	**230**	**2.97**	**1.61 – 5.46**	-	-	-	230	1.51	0.82 – 2.78	-	-	-
				
**Pro Children Study**													
				
		**N**	**OR**	**95% CI**	**N**	**OR**	**95% CI**	**N**	**OR**	**95% CI**	**N**	**OR**	**95% CI**
Liking	Decreased/stable low (1)	72	1.00	-	117	1.00	-	72	1.00	-	117	1.00	-
	Stable high – increased (0)	**118**	**3.39**	**1.80 – 6.37**	85	1.40	0.77 – 2.54	**118**	**3.23**	**1.69 – 6.15**	85	1.62	0.90 – 2.93
Knowledge	Decreased (2)	-	-	-	44	1.00	-	-	-	-	44	1.00	-
	Stable low (1) (decreased (1))	47	1.00	-	100	0.57	0.33 – 1.01	47	1.00	-	100	1.07	0.61 – 1.86
	Stable high – increased (0)	166	1.79	0.91 – 3.52	67	0.93	0.51 – 1.69	166	1.59	0.81 – 3.14	67	1.36	0.75 – 2.46
General self-efficacy	Decreased/stable low (1)	63	1.00	-	85	1.00	-	63	1.00	-	85	1.00	-
	Stable high – increased (0)	**139**	**2.19**	**1.17 – 4.10**	118	1.38	0.76 – 2.49	139	1.60	0.86 – 3.00	118	1.33	0.75 – 2.35
Modeling	Decreased/stable low (1)	110	1.00	-	95	1.00	-	110	1.00	-	95	1.00	-
	Stable high – increased (0)	80	1.16	0.64 – 2.13	**101**	**2.29**	**1.27 – 4.14**	80	1.75	0.94 – 3.25	101	1.48	0.83 – 2.64
Active encourage	Decreased/stable low (1)	100	1.00	-	86	1.00	-	100	1.00	-	86	1.00	-
	Stable high – increased (0)	**103**	**1.78**	**1.00 – 3.16**	**122**	**1.96**	**1.10 – 3.50**	103	1.79	1.00 – 3.21	122	1.63	0.92 – 2.88
Facilitation	Decreased (2)	81	1.00	-	78	1.00	-	81	1.00	-	78	1.00	-
	Stable low (1) (decreased (1))	54	0.84	0.44 – 1.60	66	0.80	0.43 – 1.46	54	0.78	0.41 – 1.49	66	0.76	0.42 – 1.38
	Stable high – increased (0)	77	1.64	0.92 – 2.94	**64**	**2.57**	**1.32 – 4.98**	77	1.77	0.97 – 3.22	**64**	**2.12**	**1.12 – 4.01**
Demand family rule	Decreased (2)	53	1.00	-	-	-	-	53	1.00	-	-	-	-
	Stable low (1) (decreased (1))	52	0.64	0.33 – 1.24	77	1.00	-	52	0.85	0.45 – 1.61	77	1.00	-
	Stable high – increased (0)	**106**	**2.63**	**1.48 – 4.68**	**138**	**3.10**	**1.66 – 5.79**	106	1.66	0.95 – 2.91	**138**	**3.06**	**1.64 – 5.68**
Allow family rule	Decreased/stable low (1)	31	1.00	-	64	1.00	-	31	1.00	-	64	1.00	-
	Stable high – increased (0)	180	0.70	0.32 – 1.54	150	1.72	0.94 – 3.16	180	1.76	0.80 – 3.91	**150**	**1.94**	**1.06 – 3.55**
Availability at home	Decreased/stable low (1)	75	1.00	-	80	1.00	-	75	1.00	-	80	1.00	-
	Stable high – increased (0)	**112**	**2.38**	**1.29 – 4.40**	115	1.47	0.79 – 2.71	112	1.38	0.76 – 2.53	**115**	**2.14**	**1.17 – 3.91**

OR – odds ration for comparison with the control group; CI, confidence interval.

Analyses are adjusted for children's age, gender, region of residence of the children (only in the Schoolgruiten study), and parental educational level

The children of the Pro Children Study who increased or maintained their relatively high vegetable intake frequency in the first time lapse were more likely to report increased levels of modeling behavior by friends and parents for eating vegetables, parental active encouragement to eat vegetables, parental facilitation of vegetables, and the family rule demanding the child to eat vegetables in the same time lapse (see Table 4). For the Schoolgruiten Project we found a significant association between positive changes or maintained relatively high levels of vegetable intake frequency and liking of vegetables (see Table 4).

### Analyses of relation B (see Figure 1)

The children who increased or maintained their relatively high fruit intake frequency later in time were more likely to have increased their liking of fruit in the previous time lapse. We found this association for both studies (see Table 4). For vegetable intake frequency in the Pro Children Study, children were more likely to report positive changes or maintained their relatively high scores in the social and physical environmental factors in the previous time lapse: parental facilitation of vegetables, family rules of eating vegetables (demanding and allowing) and availability at home of vegetables (see Table 4).

### Analyses of relation C (see Figure 1)

We found significant associations between increased or stable high fruit intake frequency in the first time lapse and increased knowledge of recommended intake levels of fruit intake later in time in the Pro Children Study. For the Schoolgruiten Project we found significant associations of increased or stable high intakes of F&V frequency in the first time lapse and increased or maintenance of high scores on liking of both F&V intake later in time.

## Discussion

The present study aimed to assess whether positive changes in or maintenance of high scores on presumed determinants of F&V intake were associated with positive changes or maintenance of favorable levels of F&V intake frequency in the same time lapse or later in time. Results indicated that behavior change was preceded by changes (or maintenance of high scores) in (some) presumed important determinants of F&V intake. Changes in these variables were even more often associated with positive changes or maintenance of favorable levels of F&V frequency intake in the same time lapse. This might be caused by the fact that most changes in determinants and intakes occurred in the first year, at least in the Pro Children Study [21], as a result of the fact that the intervention was most intensive in the first year. This results in more variation in the change in determinants and F&V intake frequency in the first time lapse than later in time, making it easier to detect associations in the first time lapse. However, the analyses within the same time lapse do not allow drawing conclusions on the direction of the relationships. The analyses including different time lapses provided this information, however, very few significant associations were observed. Furthermore, the time intervals between the different measurements might have been too long to detect whether changes in determinants precede changes in F&V intake frequency or visa versa. Therefore, more longitudinal research with shorter time intervals between the repeated measurements is needed.

Presumed determinants that predicted behavior change (= increase intake of F&V frequency) were liking of F&V, facilitation by the parents of F&V, family rules for eating F&V and availability at home of F&V. This is in accordance with social ecological behavior change theories [35] and the rationale for both intervention studies [22]. Exposure to F&V by means of the F&V scheme and taste testing were meant to influence taste preferences and subsequently to increase F&V intake frequency. Repeated exposure is an important determinant of taste preferences [15]. The family component was included to advise parents on how they could support and facilitate their children to eat more F&V and advise them to make F&V more available at home.

Although most behavioral change theories posit that changes in the presumed determinants precede changes in intakes, behavioral change theories [14] also suggest that behavior change may precede and induce changes in such factors as liking as well as perceived environmental factors. We therefore investigated if changes or maintenance of favorable levels of F&V intake frequency predicted changes in presumed determinants of F&V intake. In the Pro Children Study favorable changes in fruit intake frequency predicted increased knowledge of recommendations of fruit intake, while in the Schoolgruiten Project increased (or maintenance of favorable levels of) F&V intakes frequency predicted increased or maintenance of high scores of liking of F&V. These results may be regarded as support for a more direct relation between F&V interventions and intakes, as suggested in dual process models [14]. More research is needed to further explore such direct pathways between interventions and changes in intake levels.

As already mentioned in the background of this paper, most studies investigating correlations between F&V intake and their determinants apply cross-sectional designs that do not allow conclusions about prediction or causation. Likewise, most studies conducting mediation analyses aim at explaining intervention effects and assess changes in behavior and changes in potential mediators within the same time interval. The present findings confirm suggestions from cross-sectional studies that liking of F&V and perceived social environmental factors of F&V are indeed important predictors of F&V intake. Liking was also effected by changes in F&V intake frequency, suggesting a reciprocal relation between F&V intake and liking.

To our knowledge, no other studies investigated the association between changes in determinants and changes in F&V intake among children in different time intervals. The study of Kvaavik *et al*. [36] looked at psychosocial determinants and F&V among adults over an eight-year follow-up period and found that attitudes (men), subjective norms (men), perceived behavioral control (women) and perceived social norms (women) at age 25y predicted F&V intake at age 33y in men and women. In addition, there are some longitudinal studies available that looked at other health behaviors among adolescents and young adults. De Bourdeaudhuij *et al*. concluded that baseline psychosocial variables were poor predictors of physical activity change among 16–25 year olds, but that determinants' change scores accounted in males for 16%–19%, and in females for 7%–24% of the variance in physical activity [37]. Van De Ven *et al*. found that baseline smoking-related cognitions predicted smoking onset later in time [38]. Chang *et al*. also looked at predictive factors related to smoking onset later in time and found that peer smoking, peers offering cigarettes, alcohol use and lower protective factors in the 10th grade predicted smoking initiation by grade 12. They also found that decreases in risk factors and increases in protective factors were associated with youth smoking cessation [39]. Unfortunately, these studies used two repeated measurements in time and were therefore not able to study changes in determinants and behaviors in different time intervals. Furthermore, they did not study whether behavior change could predict changes in determinants/psychosocial factors.

Some limitations of the present study need to be addressed. First, all measurements were based on self-reported data, which may have resulted in social desirable answers. Second, the time-period between baseline and both follow-ups was rather long, and many more changes may have occurred that were not captured in the measurements. In addition, for the interpretation of the findings, we have to keep in mind that we not only studied associations between changes in determinants and changes in intake frequency. Children that maintained high scores on determinants or kept favorable levels of intake of F&V were also included in the category representing positive change. This might have blurred the findings. However, sensitivity analyses excluding the children that showed no change in either the determinants or intake, did not result in different effect estimates.

## Conclusion

In accordance with behavior change theories, the present study provides some evidence that behavior change (increased intake or maintenance of favorable levels of F&V frequency) was preceded by changes in or maintenance of high scores of (some) presumed determinants of F&V intakes, both in the Pro Children Study and in the Schoolgruiten Project. Determinants of F&V intake that appear to be important to induce behavior change were liking of F&V, facilitation by the parents of F&V, family rules for eating F&V and availability at home of F&V. Furthermore, changes in F&V intake frequency also induced changes in liking of F&V and knowledge of recommended intake levels of fruit.

## Competing interests

The authors declare that they have no competing interests.

## Authors' contributions

NIT collected and analysed the data and drafted the manuscript. SJtV and JB participated in the study design and provided critical revision of the manuscript. All three authors have read and approved the final manuscript.
